# Angiotensin converting enzyme inhibition increases ADMA concentration in patients on maintenance hemodialysis – a randomized cross-over study

**DOI:** 10.1186/s12882-015-0162-x

**Published:** 2015-10-22

**Authors:** Jorge L. Gamboa, Mias Pretorius, Katie C. Sprinkel, Nancy J. Brown, T. Alp Ikizler

**Affiliations:** Division of Clinical Pharmacology, Department of Medicine, Vanderbilt University Medical Center, 2222 Pierce Avenue, 561-B PRB, Nashville, TN 37232 USA; Division of Nephrology, Department of Medicine, Vanderbilt University Medical Center, Nashville, TN USA; Department of Anesthesiology, Vanderbilt University Medical Center, Nashville, TN USA

**Keywords:** Asymmetric dimethylarginine, Hemodialysis, Angiotensin converting enzyme inhibitors, Angiotensin receptor blocker

## Abstract

**Background:**

Endothelial dysfunction occurs in patients with end-stage renal disease (ESRD) and is associated with increased cardiovascular morbidity and mortality. Asymmetric dimethylarginine (ADMA) contributes to endothelial dysfunction in ESRD. In the general population, angiotensin-converting enzyme (ACE) inhibitors and angiotensin receptor blockers (ARBs) decrease ADMA levels, but no study has compared the effect of these drugs in patients with ESRD on maintenance hemodialysis (MHD).

**Methods:**

We evaluated the effect of 1-week treatment with ramipril (5 mg/d), valsartan (160 mg/d), and placebo on ADMA levels in 15 patients on MHD in a double-blind, placebo-controlled, three x three cross-over study.

**Results:**

We found that ADMA levels were increased at baseline and throughout the dialysis session during ramipril treatment (*p* < 0.001 compared to both, placebo and valsartan). Ramipril did not increase ADMA levels in a study of patients without ESRD, suggesting that factors related to ESRD or hemodialysis contribute to the ACE inhibitor-induced increase in ADMA. We have previously shown that ACE inhibition increases bradykinin (BK) levels during hemodialysis. We therefore evaluated the effect of bradykinin on ADMA production in A549 cells; a cell line that expresses BK receptors. Incubation with BK increased intracellular ADMA concentration through BK B2-receptor stimulation.

**Conclusion:**

These data indicate that short-term ACE inhibition increases ADMA in patients on MHD whereas ARBs do not. *In vitro* studies further suggest that this may occur through BK-mediated increase in ADMA production during ACE inhibition.

**Trial registration:**

Clinicaltrials.gov NCT00732069 August 6 2008 and NCT00607672 February 4 2008

**Electronic supplementary material:**

The online version of this article (doi:10.1186/s12882-015-0162-x) contains supplementary material, which is available to authorized users.

## Background

Patients undergoing maintenance hemodialysis (MHD) exhibit accelerated atherosclerosis and are at high risk of developing cardiovascular events, even after controlling for traditional risk factors [1–3]. Certain non-traditional factors, such as oxidative stress and inflammation, have been implicated in the pathogenesis of atherosclerosis of end-stage renal disease (ESRD) [4]. We and others have described that patients on MHD have increased markers of oxidative stress and inflammation, [5–7] which are worsened by loss of kidney function and to some extent with hemodialysis therapy.

Endothelial dysfunction is also commonly present in patients with ESRD and is associated with an increased risk of atherosclerotic events [8]. Increased levels of asymmetric dimethylarginine (ADMA), a potent inhibitor of endothelial nitric oxide synthase, can contribute to endothelial dysfunction. ADMA levels correlate with atherosclerosis and predict morbidity and mortality in patients with ESRD [9, 10]. Arginine methylation occurs as a post-translational modification of proteins [11]. After protein degradation, symmetric and asymmetric methylarginines are released and transported out of the cell through cationic amino-acid transporters [12]. Elimination of symmetric dimethylarginine (SDMA) occurs primarily through glomerular filtration, whereas ADMA elimination depends on enzymatic degradation. Two enzymatic pathways are important in the elimination of ADMA: dimethyl-arginine dimethylamino-hydrolases (DDAH), enzymes expressed in brain, kidney and liver, the alanine glyoxylate aminotransferase 2 (AGXT2), a mitochondrial enzyme highly expressed in the kidney and liver [13–15]. Regardless of the mechanism of elimination, plasma levels of SDMA and ADMA are increased in patients with chronic kidney disease (CKD) and ESRD [11, 16].

ADMA may also impair endothelial function by activating the renin-angiotensin system [17]. Conversely angiotensin II increases ADMA levels through activation of the NADPH oxidase [18]. Previous studies have shown that treatment with angiotensin-converting enzyme (ACE) inhibitors and angiotensin receptor blockers (ARBs) reduce ADMA levels in the general population [19–22]. To date, no study has compared the effects of ARBs or ACE inhibitors on ADMA levels in patients with ESRD. Based on studies in the general population, we hypothesized that short-term administration of either an ACE inhibitor or an ARB would lower ADMA levels in patients with ESRD undergoing MHD. We also hypothesized that these effects would be more noticeable during hemodialysis due to concurrent activation of the inflammatory response. In order to test this hypothesis, we measured ADMA levels in a three x three crossover, randomized double blind, placebo-controlled study designed to evaluate the effects of 1-week treatment of either ACE inhibitor or ARB.

## Methods

### Study population

Details of the study have been previously described (NCT00732069, clinicaltrials.gov) [5]. The study was approved by the Vanderbilt University Institutional Review Board. Briefly, adult subjects (18 years or older) with ESRD undergoing MHD (for at least 6 months) 3 times per week were included in the study. Patients were clinically stable with a pre-dialysis potassium levels less than 5.5 mmol/L and adequately dialyzed (kt/V >1.2) with polysulphone membranes. Exclusion criteria for the study were the following: history of active connective tissue disease, acute infection within 1 month prior to the study, advanced liver disease, gastrointestinal dysfunction requiring parental nutrition or active malignancy, medications such as immunosuppressive drugs within 1 month prior to the study, use of anti-inflammatory medications other than aspirin less than 325 mg per day, use of vitamin E at a dose higher than 60 IU per day or vitamin C higher than 500 mg per day; history of myocardial infarction or cerebrovascular event within 3 months prior to the study, or ejection fraction lower than 40 %, pregnancy, breastfeeding, history of ACE inhibitor-associated angioedema, inability to discontinue ACE inhibitors or ARBs. Twenty patients participated in the study and signed informed consent. Fifteen patients completed the whole study. One subject voluntarily withdrew the study after being consented, and three participants were excluded due to exclusion criteria (uncontrollable hypertension, hypotension, and hyperkalemia). One subject was withdrawn from the study because of an event (cerebral ischemic stroke) that was not study related. Participants were recruited between October 2008 and January 2010; the study ended on March 2010. Further details of the study population have been published previously [5]. Additional file 1: Figure S1 summarizes the patient enrollment process.

### Study protocol

The study was a three x three cross-over design, randomized, double-blind, placebo-controlled. Patients received ramipril (King Pharmaceutical, Bristol, TN), valsartan (Novartis Pharmaceutical, East Hanover, NJ) or placebo for 7 days. After a washout period of 3 weeks, patients received another treatment until every patient completed three treatment arms. The order of treatment was randomized using a three x three orthogonal Latin square design, as specified in Jones and Kenward [23]. Treatment was dispensed by Vanderbilt Investigational Drug Services. Ramipril was administered for 2 days at 2.5 mg/d followed by 5 mg/d the following 5 days. Valsartan was given at 80 mg/d for 2 days, followed by 160 mg/d the following 5 days. On the seventh day of treatment, patients took the study medication early in the morning and arrived to the Clinical Research Center (CRC) at Vanderbilt University Medical Center for a regular session of hemodialysis. Blood samples were collected before the beginning of dialysis, 30 min, 1 h later, at the end of dialysis (4 h) and 2 h after the end of hemodialysis.

### ADMA levels

Blood samples were collected in tubes containing EDTA. After centrifugation, plasma was immediately collected and stored at −80 °C until processing for analysis. Levels of ADMA, SDMA, and arginine were measured by high performance liquid chromatography (HPLC) with fluorescence detection and using monomethyl-arginine as internal standard. Plasma samples were separated using solid-phase extraction, and analytes were derivatized with ortho-phthalaldehyde containing 3-mercaptopropionic acid, as previously described [24]. Methylarginines levels were also measured in plasma obtained from participants in clinical trial NCT00607672 of subjects undergoing cardiac surgery, who were randomized to one week treatment with ramipril, candesartan or placebo prior to surgery.

#### Markers of inflammation and oxidative stress

Serum IL-6 concentrations were measured using Luminex immunoassay. Monocyte chemoattractant protein 1 levels were measured using a commercial kit (Linco Research, St. Charles, MO) according to the manufacturer instructions. Soluble CD40 ligand concentrations were measured in plasma using an ELISA kit (Quantikin, R&D Systems, Minneapolis, MN). We measured these inflammatory markers in our previous study [5]. F2-isoprostanes, which is a stable and reliable marker of lipid peroxidation, were measured in plasma using ion gas-chromatophragy mass spectroscopy (GC/MS), as previously described [25, 26].

### Cell culture

Human alveolar adenocarcinoma cells (A549) from passages four to six were used for the experiments. This cell line was used due to the constitutively expression of bradykinin receptors. Cells were cultured in DMEM medium supplemented with 10 % of fetal bovine serum at 37 °C. Cells were grown to confluence in 6-well plates and incubated in 2 ml of serum-free medium for 24 h. Cells were treated with vehicle, bradykinin (20 nM), bradykinin + the B2 receptor antagonist HOE-140 (1 μM), or bradykinin + the B1 receptor antagonist Lys (Des-Arg9-Leu8) bradykinin (1 μM). At the end of the treatment, culture medium was collected, centrifuged at 2500 g for 10 min, and stored at −80 °C. Cells were washed three times with PBS, scraped into PBS, and centrifuged for 5 min at 600xg. Cells were re-suspended in 250 μl of ice-cold sodium phosphate (100 mM, pH 6.5) and sonicated on ice. Cell lysate (220 μl) was mixed with 220 μl of perchloric acid (1.2 M), centrifuged at 2000 g for 10 min at 4 °C, and the supernatant collected and stored at −80 °C until further processing. Data was normalized by total protein content in the cells lysate and measured with the Pierce® BCA kit (Thermo Scientific, Rockford IL) according the manufacturer protocol.

### Statistical analysis

Sample size was calculated based on the effect of treatment on IL-6 concentrations with 95 % power to detect a 30 % difference between ACEi and ARB. Data was presented as mean +/− standard error of the mean (SEM). A linear mixed effect model was used to compare the effect of treatment (ACEi, ARB, or placebo) during hemodialysis (time) on ADMA, SDMA, arginine and arginine to ADMA ratios. The model was selected based on the lower Akaike’s information criteria. ADMA levels and arginine-to-ADMA ratios were correlated with markers of inflammation and oxidative using Spearman’s rank correlation test. A two side *P* < 0.05 was considered significant. Data analysis was performed using SPSS software (v. 20.0, IBM).

## Results

### Baseline patient demographics

Fifteen subjects were included in the study. Table 1 summarizes the demographic characteristic of the hemodialysis study participants. Causes of ESRD were diabetes mellitus (28 %, *n* = 4), hypertension (60 %, *n* = 9), non-steroidal anti-inflammatory drug (NSAID)-induced glomerulonephritis (6 %, *n* = 1), and unknown (6 %, *n* = 1).

**Table 1:** Baseline characteristics

Parameter	Value
Age (yr)	50.5 ± 3.1
Gender (male:female)	7:8
Race (African-American: Caucasian: Hispanic)	(11:2:2)
Smoker (yes:no)	(5:10)
Hypertension (yes:no)	15:0
Previous ACEi or ARB use (yes:no)	3:12
BMI (kg/m^2^)	30.2 ± 2.0
Calcium x Phosphate product (mg^2^/dl^2^)	53.3 ± 3.3
Hemoglobin (g/dl)	11.9 ± 0.3
Erythropoietin dose (units)	5111.1 ± 1488.4
Heparin dose (units)	4800 ± 579.0

Data are presented as mean ± SEM

### Effect of treatment on blood pressure and heart rate

Systolic and diastolic blood pressure prior to the beginning of dialysis was not different among the treatment arms (Table 2). Heart rate was also comparable among the treatment arms (Table 2).

**Table 2:** Blood pressure and heart rate prior to the beginning of hemodialysis

Parameter	Placebo	Ramipril	Valsartan
SBP	134.3 ± 7.4	137.4 ± 7.4	141.1 ± 1.7
DBP	77.0 ± 3.5	75.5 ± 3.7	74.4 ± 3.2
Heart rate	76.7 ± 4.1	75.5 ± 4.0	75.5 ± 3.7

Data are presented as mean ± SEM

### Effect of hemodialysis and treatment on methylarginines

**Fig. 1:**
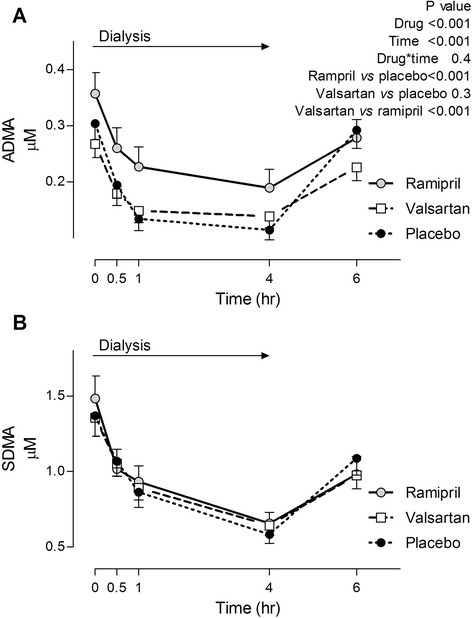
Effect of 1 week treatment with ramipril, valsartan and placebo on (**a**) asymmetric dimethylarginine (ADMA) and (**b**) symmetric dimethylarginine in patients with end-stage renal disease during hemodialysis

ADMA and SDMA levels decreased by 30 min, reached a nadir at 1 h after the initiation of hemodialysis (Fig. 1), and remained lower at the end of hemodialysis (4 h). In contrast, SDMA reach the lowest level at the end of hemodialysis. Methylarginines levels increased 2 h after the end of hemodialysis but remained lower than baseline levels (14 and 27 % lower than baseline for ADMA and SDMA respectively). ADMA levels were significantly higher at baseline and throughout dialysis during ramipril treatment (Fig. 1) compared to treatment with either placebo or valsartan. Treatment with ramipril or valsartan did not affect SDMA levels.

### Effects of hemodialysis and treatment on arginine and arginine to ADMA ratio

**Fig. 2:**
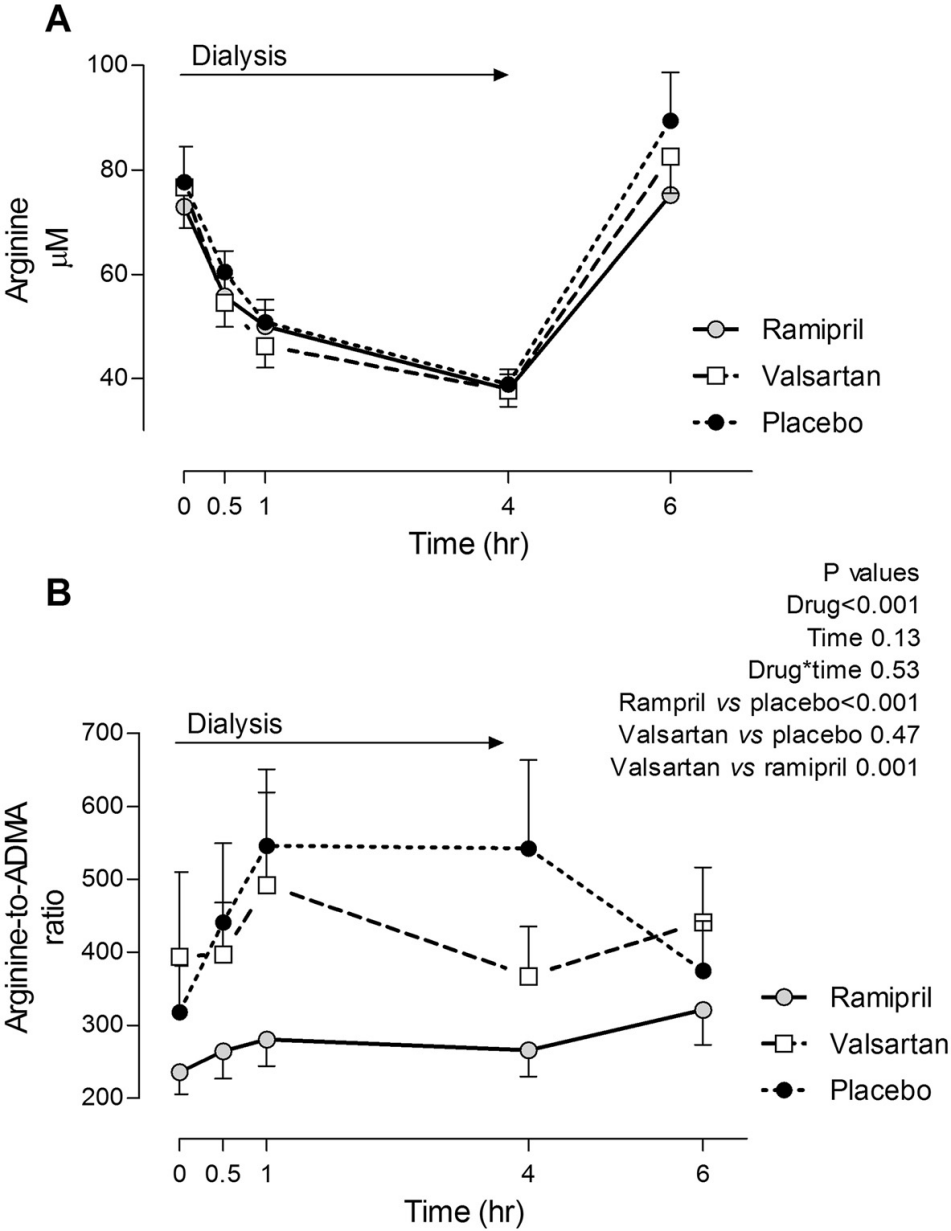
Effect of 1 week treatment with ramipril, valsartan and placebo on (**a**) arginine levels and (**b**) ratios of arginine- to-asymmetric dimethylarginine (ADMA) in patients with end-stage renal disease during hemodialysis

Arginine levels also decreased during hemodialysis, reaching the lowest level at the end of hemodialysis; 2 h later arginine levels returned to pre-dialysis levels (Fig. 2). Treatment with ramipril or valsartan did not affect arginine levels (Fig. 2). Arginine-to-ADMA ratios were lower in patients treated with ramipril at the beginning of hemodialysis and did not change during dialysis (Fig. 2). Conversely, treatment with either valsartan or placebo increased the arginine-to-ADMA ratio during hemodialysis.

### Correlation of ADMA with markers of inflammation and oxidative stress

ADMA levels correlated positively with markers of inflammation including interleukin-6 (IL-6; *ρ* = 0.23, *p* = 0.001), monocyte chemoattractant protein-1 (MCP-1; *ρ* = 0.27, *p* < 0.001), and soluble CD 40 ligand (sCD40L; *ρ* = 0.22, *p* = 0.001). The arginine-to-ADMA ratio correlated negatively with the same markers of inflammation; IL-6 (*ρ* = −0.26, *p* < 0.001), MCP-1 (*ρ* = −0.29, *p* < 0.001), and sCD40L (*ρ* = −0.34, *p* < 0.001). The ratio also correlated negatively with F2-Isoprostanes (*ρ* = −0.31, *p* < 0.001), a marker of oxidative stress.

### Effect of ramipril on methylarginines levels in patients with no history of ESRD

**Fig. 3:**
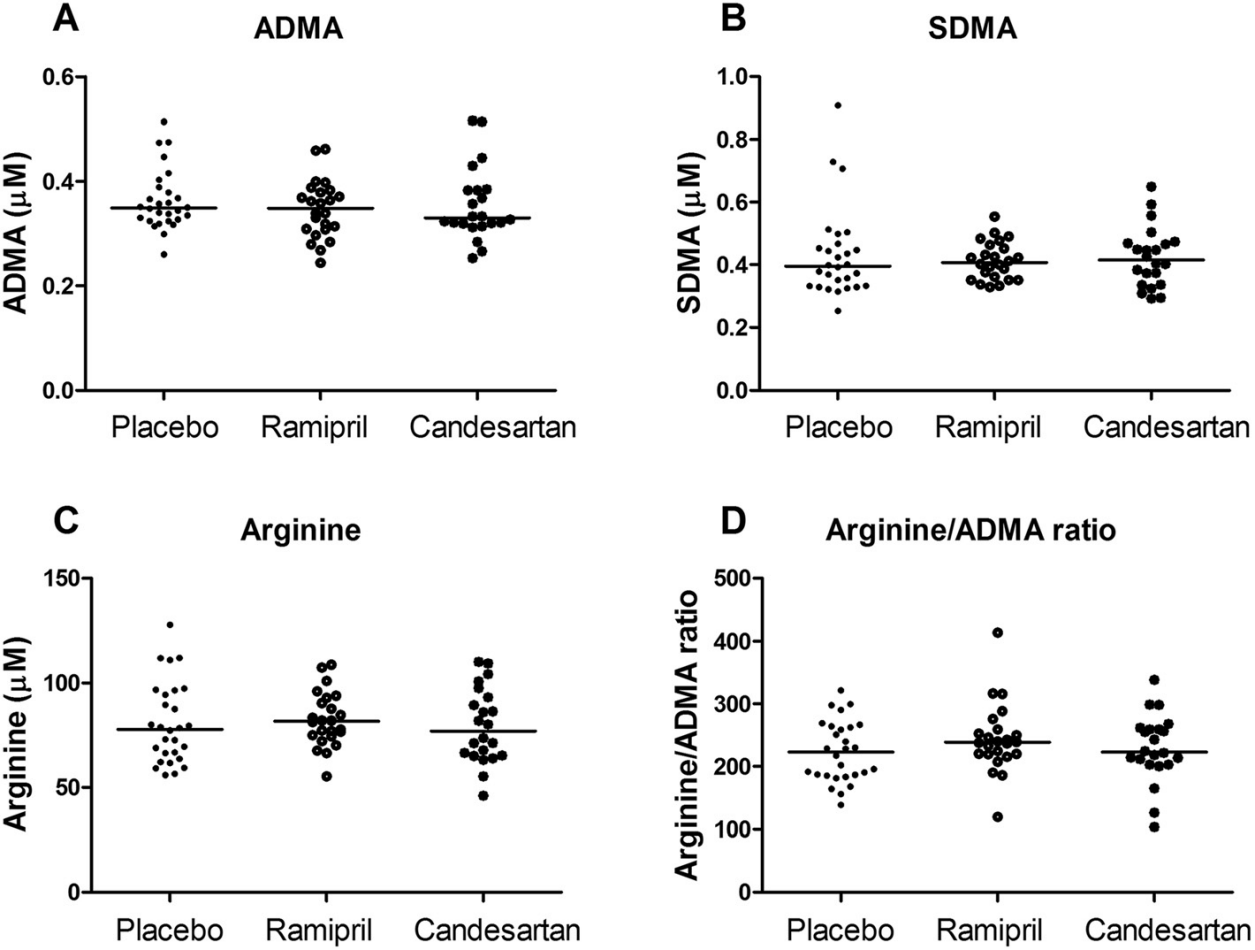
Effect of 1 week treatment with ramipril, candesartan and placebo on (**a**) asymmetric dimethylarginine (ADMA), **b** symmetric dimethylarginine, **c** arginine levels, and (**d**) arginine-to-asymmetric dimethylarginine (ADMA) ratios in patients with no history of end-stage renal disease

To evaluate if ramipril increases ADMA levels in patients with no history of ESRD, methylarginines levels were measured in subjects that were treated for 1 week with ramipril, candesartan, or placebo (NCT00607672) [27]. Additional file 2: Table S1 summarizes the baseline demographic characteristics among the three treatment groups. One- week treatment with ramipril did not affect levels of ADMA, SDMA, or arginine (Fig. 3).

### Bradykinin increases ADMA level in human alveolar adenocarcinoma cells

**Fig. 4:**
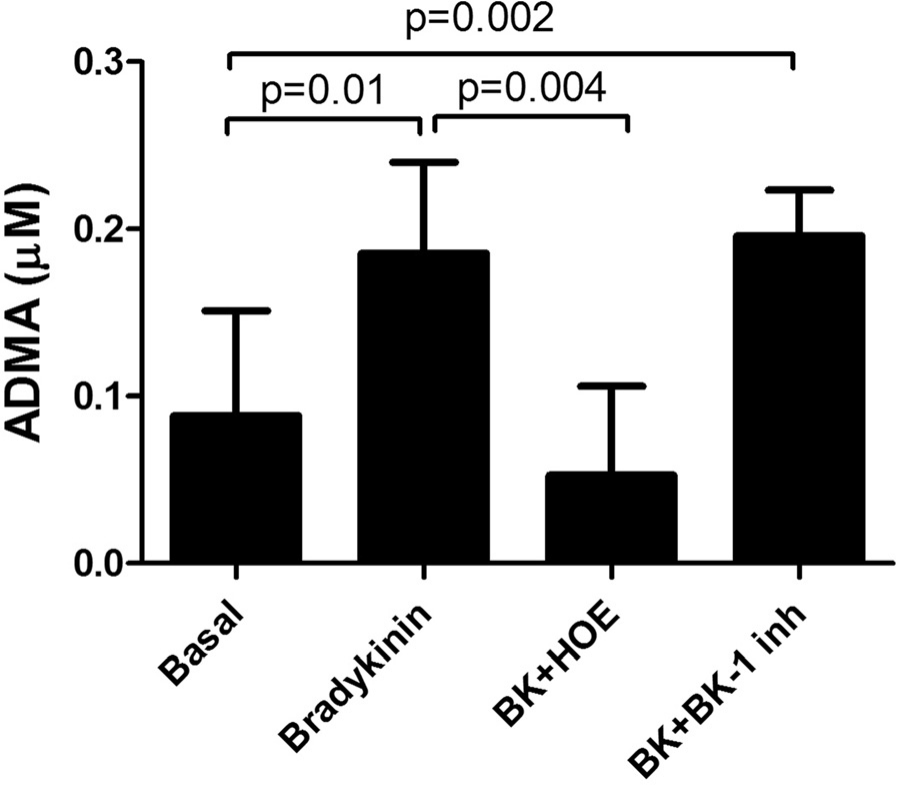
Effect of bradykinin (BK, 20nM) incubation on intracellular asymmetric dimethylarginine (ADMA) levels in human adenocarcinoma cell (A549). HOE stands for HOE-140, a bradykinin B2 receptor inhibitor (1 μM). Bradykinin B1 inhibition (B1 inh) was performed using the B1 receptor antagonist Lys (Des-Arg9-Leu8) BK (1 μM). Bars represent mean ± standard deviation

ACE inhibitors, but not ARBs, increase bradykinin levels. Thus, we evaluated the effect of bradykinin on ADMA in human alveolar adenocarcinoma cells (A549), a cell line that expresses bradykinin receptors. A549 cells were incubated in the presence bradykinin and bradykinin receptors inhibitors. ADMA levels increased with incubation of bradykinin. Co-incubation with bradykinin B1 receptor inhibitor had no role in decreasing ADMA, whereas bradykinin B2 receptor inhibition reduced intracellular ADMA levels (Fig. 4).

## Discussion

This study examined the effects of ACE inhibitor and ARB on methylarginines in patients undergoing MHD. We found that short-term treatment with ramipril (1 week) increased ADMA levels and decreased the arginine-to-ADMA ratio compared to placebo and valsartan in patients undergoing MHD. Studies *in vitro* suggest that increase in ADMA production occur through stimulation of bradykinin B2 receptors.

ADMA levels are increased in patients undergoing MHD and correlate with all-cause mortality [9]. In addition, ADMA levels correlate positively with markers of atherosclerosis such as carotid intima-media thickness, left ventricular hypertrophy, and cardiovascular events [9, 10, 28, 29]. Arginine and ADMA are substrate and inhibitor of endothelial nitric oxide synthase, respectively; thus the ratio of arginine to ADMA may modify the enzyme activity [30]. Accordingly, a lower arginine to ADMA ratio has been also been associated with all-cause mortality and carotid intima-media thickness in the general population [31–33]. While the pathogenic role of ADMA in the cardiovascular disease is somewhat unclear, it has been shown to induce endothelial dysfunction, which is involved in the initiation and progression of atherosclerosis [34]. Another possible factor that can contribute to increase cardiovascular risk is the over-activation of the sympathetic nervous system by ADMA. Experiments in humans have shown that nitric oxide inhibition results in the activation of the sympathetic nervous system [35], a known risk factor cardiovascular disease. In fact, ADMA levels strongly correlates with norepinephrine levels in patients with ESRD [36].

We did not find any effect of either ramipril or candersatan on ADMA levels in patients with preserved kidney function. But previous studies demonstrated that both ACE inhibitors and ARBs reduce ADMA levels in patients without kidney disease [19–22]. The duration of the treatment, one week *vs*. several weeks and months, may explain why neither ACE inhibitor nor ARB reduce ADMA levels in our study. Longer treatment with ACE inhibitors or ARBs may decrease ADMA by decreasing angiotensin II effects. Angiotensin II increases ADMA levels in smooth muscle cells by direct stimulation of AT1 receptors and by increasing oxidative stress [18]. The latter is probably mediated through the production of reactive oxygen species by NADPH oxidase [37, 38]. Only one previous study in patients on MHD showed that 6-week treatment with valsartan or amlodipine reduced ADMA and SDMA [39]. Findings from this study were attributed to a reduction in shear stress, which has been shown to increase ADMA release from endothelial cells [40]. In comparison, we did not find reduction in ADMA after 1 week treatment with valsartan. Again, it is possible that a longer treatment with ARB may have an effect on ADMA levels. Nevertheless, our study is the first one to compare the effect of ACE inhibitors and ARBs on ADMA levels in patients on MHD.

In this study we found that ramipril, an ACE inhibitor, but not valsartan, an ARB, increased ADMA level. We found also that ramipril did not increase ADMA level in patients without ESRD, suggesting that other factors pertaining to ESRD, particularly hemodialysis may contribute to the ramipril-induced increase in ADMA levels. A potential explanation as to why ACE inhibitors and ARBs differ in their effect on ADMA levels could be that these medications also differ in their effect on the degradation of bradykinin. Specifically, ACE inhibitors increase bradykinin bioavailability, [41] whereas ARBs does not. We have previously shown that ramipril treatment increased bradykinin levels during hemodialysis [5]. Previous studies have shown that bradykinin increases reactive oxygen species production through stimulation of NADPH oxidases [42]. NADPH activation increases ADMA levels by increasing protein methylation and inhibiting ADMA degradation, [18, 43] which are both mediated by reactive oxygen species. Thus, it is possible that ramipril increases ADMA levels by increasing bradykinin and oxidative stress. Our *in vitro* studies support this hypothesis. We found that bradykinin contributes to ADMA production in human adenocarcinoma cells. Importantly, we also found that incubation with the bradykinin B2 receptor antagonist HOE-140 reduces intracellular ADMA levels. Bradykinin B1 receptor blockage does not play a role in decreasing ADMA levels suggesting that bradykinin-dependent ADMA production occurs through bradykinin B2 receptor stimulation. A possible mechanism that has not been explored is the role of bradykinin on DDAH, the enzyme responsible for ADMA degradation. Bradykinin may increase ADMA levels by decreasing DDAH activity.

We also found that markers of inflammation positively correlate with ADMA levels and inversely with the arginine-to-ADMA ratio. The latter also has an inverse correlation with F2-Isoprostanes. These findings are in agreement with previous studies that showed the correlation between ADMA and inflammatory markers (C-reactive protein and IL-6) [10, 44]. We previously described that ramipril has a greater pro-inflammatory effect in patients with ESRD [5]. Given the interrelation between ADMA and inflammation, it is not surprising that ACE inhibitors also increase ADMA levels in patients on MHD.

Our findings may some interesting implications. First and foremost, these data could explain the relative lack of efficacy of ACE inhibitors in improving cardiovascular outcomes in patients with ESRD, at least to some extent. To date, there are no clear-cut clinical guidelines for the use of ACE inhibitor or ARBs in patients with ESRD, mostly due to lack of randomized clinical trials. If anything, limited epidemiological data on this subject indicate that use of ACE inhibitor could be detrimental in patients with ESRD, at least when used in combination [45]. Accordingly, further studies are necessary to examine the appropriate use of these potentially useful classes of cardiovascular medications. In addition, these data demonstrate that patients with ESRD, particularly ones on MHD behave differently in response to medications commonly used in the general populations. Accordingly, one should not simply assume that what has been observed in response to a medication in the general population can be safely extrapolated to patients with ESRD. Finally, our data once again highlight the importance of the so-called non-traditional cardiovascular risk factors in patients with ESRD. While this particular study focused on ACE inhibitors and ARBs, other interventions that modulate one or more of these metabolic derangements should be aggressively studied in patients with ESRD.

The strengths of the study are its design, a cross-over study that eliminates the within-subject variation, and the biological relevance of the findings. Study limitations include the short-duration of treatment (1 week) and a small sample size. It is notable that we nevertheless had sufficient statistical power to demonstrate significant differences. Finally, the levels of methylarginines are somewhat different from other published studies although there is a wide variability in terms of blood concentrations of ADMA and SDMA in ESRD patient population. In this study, we only used high-flux dialyzers, which may affect the removal of methylarginines. Although a previous study showed the type of dialyzer does not affect plasma ADMA removal, [46] the choice of dialyzer could explain the differential pre-dialysis levels in methylarginines compared to other published studies [9, 10, 47]. Nevertheless, use of same subjects as their own controls in this study should compensate for any difference on baseline level of methylarginines and the comparative effects of ACE inhibitor and ARB administered throughout the study.

## Conclusions

In conclusion, we found that short-term ACE inhibitor and ARB administration differentially affects ADMA levels and the arginine-to-ADMA ratio in patients undergoing MHD. These parameters also correlate with markers of inflammation and oxidative stress. While these data suggest that ARB therapy may be a better treatment option for improving non-traditional cardiovascular risk factors in patients on MHD, further studies are required to determine the real therapeutic benefit of these interventions in order to reduce atherosclerotic burden in this patient population.

## Additional files

Additional file 1: Figure S1. CONSORT Flow Diagram. (DOCX 43 kb) Additional file 2: Table S1. Baseline characteristics of patients with no history of CKD. (DOCX 14 kb)

## Abbreviations

ADMA: Asymmetric dimethylarginine
SDMA: Symmetric dimethylarginine
ACE: Angiotensin converting enzyme
ARB: Angiotensin receptor blocker
MHD: Maintenance hemodialysis
ESRD: End-stage renal disease
BK: Bradykinin

## References

[1] Foley RN, Parfrey PS, Sarnak MJ (1998). Epidemiology of cardiovascular disease in chronic renal disease. J Am Soc Nephrol.

[2] Foley RN, Parfrey PS, Sarnak MJ (1998). Clinical epidemiology of cardiovascular disease in chronic renal disease. Am J Kidney Dis.

[3] Sarnak MJ, Levey AS (1999). Epidemiology of Cardiac Disease in Dialysis Patients. Semin Dial.

[4] Himmelfarb J (2009). Uremic toxicity, oxidative stress, and hemodialysis as renal replacement therapy. Semin Dial.

[5] Gamboa JL, Pretorius M, Todd-Tzanetos DR, Luther JM, Yu C, Ikizler TA (2012). Comparative Effects of Angiotensin-Converting Enzyme Inhibition and Angiotensin-Receptor Blockade on Inflammation during Hemodialysis. J Am Soc Nephrol.

[6] Ikizler TA, Morrow JD, Roberts LJ, Evanson JA, Becker B, Hakim RM (2002). Plasma F2-isoprostane levels are elevated in chronic hemodialysis patients. Clin Nephrol.

[7] Handelman GJ, Walter MF, Adhikarla R, Gross J, Dallal GE, Levin NW (2001). Elevated plasma F2-isoprostanes in patients on long-term hemodialysis. Kidney Int.

[8] Stenvinkel P (2003). Interactions between inflammation, oxidative stress, and endothelial dysfunction in end-stage renal disease. J Ren Nutr.

[9] Zoccali C, Bode-Böger SM, Mallamaci F, Benedetto FA, Tripepi G, Malatino LS (2001). Plasma concentration of asymmetrical dimethylarginine and mortality in patients with end-stage renal disease: a prospective study. Lancet.

[10] Zoccali C, Benedetto FA, Maas R, Mallamaci F, Tripepi G, Salvatore Malatino L (2002). Asymmetric Dimethylarginine, C-Reactive Protein, and Carotid Intima-Media Thickness in End-Stage Renal Disease. J Am Soc Nephrol.

[11] Leone A, Moncada S, Vallance P, Calver A, Collier J (1992). Accumulation of an endogenous inhibitor of nitric oxide synthesis in chronic renal failure. Lancet.

[12] Closs EI, Basha FZ, Habermeier A, Förstermann U (1997). Interference of L-Arginine Analogues with L-Arginine Transport Mediated by the y + Carrier hCAT-2B. Nitric Oxide.

[13] Schwedhelm E, Boger RH (2011). The role of asymmetric and symmetric dimethylarginines in renal disease. Nat Rev Nephrol.

[14] Pope AJ, Karuppiah K, Cardounel AJ (2009). Role of the PRMTΓÇôDDAHΓÇôADMA axis in the regulation of endothelial nitric oxide production. Pharmacol Res.

[15] Rodionov RN, Jarzebska N, Weiss N, Lentz SR (2014). AGXT2: a promiscuous aminotransferase. Trends Pharmacol Sci.

[16] Anderstam B, Katzarski K, Bergström J (1997). Serum levels of *N*^*G*^, *N*^*G*^-dimethyl-L-arginine, a potential endogenous nitric oxide inhibitor in dialysis patients. J Am Soc Nephrol.

[17] Veresh Z, Racz A, Lotz G, Koller A (2008). ADMA Impairs Nitric Oxide−Mediated Arteriolar Function Due to Increased Superoxide Production by Angiotensin II−NAD(P)H Oxidase Pathway. Hypertension.

[18] Luo Z, Teerlink T, Griendling K, Aslam S, Welch WJ, Wilcox CS (2010). Angiotensin II and NADPH Oxidase Increase ADMA in Vascular Smooth Muscle Cells. Hypertension.

[19] Ito A, Egashira K, Narishige T, Muramatsu K, Takeshita A (2001). Renin-Angiotensin System is Involved in the Mechanism of Increased Serum Asymmetric Dimethylarginine in Essential Hypertension. Jpn Circ J..

[20] Abbott K, Trespalacios F, Agodoa L, Ahuja T (2003). HIVAN and medication use in chronic dialysis patients in the United States: analysis of the USRDS DMMS Wave 2 study. BMC Nephrol.

[21] Napoli C, Sica V, de Nigris F, Pignalosa O, Condorelli M, Ignarro LJ (2004). Sulfhydryl angiotensin-converting enzyme inhibition induces sustained reduction of systemic oxidative stress and improves the nitric oxide pathway in patients with essential hypertension. Am Heart J.

[22] Willoughby SR, Rajendran S, Chan WP, Procter N, Leslie S, Liberts EA (2012). Ramipril Sensitizes Platelets to Nitric Oxide: Implications for Therapy in High-Risk Patients. J Am Coll Cardiol.

[23] Jones B, Kenward MG (2003). Design and Analysis of Crossover Trials.

[24] de Jong S, Teerlink T (2006). Analysis of asymmetric dimethylarginine in plasma by HPLC using a monolithic column. Anal Biochem.

[25] Milne GL, Sanchez SC, Musiek ES, Morrow JD (2007). Quantification of F2-isoprostanes as a biomarker of oxidative stress. Nat Protocols.

[26] Morrow JD, Hill KE, Burk RF, Nammour TM, Badr KF, Roberts LJ (1990). A series of prostaglandin F2-like compounds are produced in vivo in humans by a non-cyclooxygenase, free radical-catalyzed mechanism. Proc Natl Acad Sci U S A.

[27] Billings FT, Balaguer JM, Yu C, Wright P, Petracek MR, Byrne JG (2012). Comparative Effects of Angiotensin Receptor Blockade and ACE Inhibition on the Fibrinolytic and Inflammatory Responses to Cardiopulmonary Bypass. Clin Pharmacol Ther.

[28] Zoccali C, Mallamaci F, Maas R, Benedetto FA, Tripepi G, Malatino LS (2002). Left ventricular hypertrophy, cardiac remodeling and asymmetric dimethylarginine (ADMA) in hemodialysis patients. Kidney Int.

[29] Kumagai H, Sakurai M, Takita T, Maruyama Y, Uno S, Ikegaya N (2006). Association of Homocysteine and Asymmetric Dimethylarginine With Atherosclerosis and Cardiovascular Events in Maintenance Hemodialysis Patients. Am J Kidney Dis.

[30] Bode-Böger SM, Scalera F, Ignarro LJ (2007). The l-arginine paradox: Importance of the l-arginine/asymmetrical dimethylarginine ratio. Pharmacol Ther.

[31] Notsu Y, Yano S, Shibata H, Nagai A, Nabika T (2015). Plasma arginine/ADMA ratio as a sensitive risk marker for atherosclerosis: Shimane CoHRE study. Atherosclerosis.

[32] Pizzarelli F, Maas R, Dattolo P, Tripepi G, Michelassi S, D’Arrigo G (2013). Asymmetric dimethylarginine predicts survival in the elderly. Age.

[33] Böger RH, Sullivan LM, Schwedhelm E, Wang TJ, Maas R, Benjamin EJ (2009). Plasma Asymmetric Dimethylarginine and Incidence of Cardiovascular Disease and Death in the Community. Circulation.

[34] Davignon J, Ganz P (2004). Role of Endothelial Dysfunction in Atherosclerosis. Circulation.

[35] Young CN, Fisher JP, Gallagher KM, Whaley-Connell A, Chaudhary K, Victor RG (2009). Inhibition of nitric oxide synthase evokes central sympatho-excitation in healthy humans. J Physiol.

[36] Mallamaci F, Tripepi G, Maas R, Malatino L, Böger R, Zoccali C (2004). Analysis of the Relationship between Norepinephrine and Asymmetric Dimethyl Arginine Levels among Patients with End-Stage Renal Disease. J Am Soc Nephrol.

[37] Griendling KK, Minieri CA, Ollerenshaw JD, Alexander RW (1994). Angiotensin II stimulates NADH and NADPH oxidase activity in cultured vascular smooth muscle cells. Circ Res.

[38] Pagano PJ, Chanock SJ, Siwik DA, Colucci WS, Clark JK (1998). Angiotensin II Induces p67phox mRNA Expression and NADPH Oxidase Superoxide Generation in Rabbit Aortic Adventitial Fibroblasts. Hypertension.

[39] Aslam S, Santha T, Leone A, Wilcox C (2006). Effects of amlodipine and valsartan on oxidative stress and plasma methylarginines in end-stage renal disease patients on hemodialysis. Kidney Int.

[40] Osanai T, Saitoh M, Sasaki S, Tomita H, Matsunaga T, Okumura K (2003). Effect of Shear Stress on Asymmetric Dimethylarginine Release From Vascular Endothelial Cells. Hypertension.

[41] Swartz SL, Williams GH, Hollenberg NK, Levine L, Dluhy RG, Moore TJ (1980). Captopril-induced Changes in Prostaglandin Production: Relationship to Vascular Responses in Normal Man. J Clin Invest.

[42] Larsen BT, Bubolz AH, Mendoza SA, Pritchard KA, Gutterman DD (2009). Bradykinin-induced dilation of human coronary arterioles requires NADPH oxidase-derived reactive oxygen species. Arterioscler Thromb Vasc Biol.

[43] Wilcox CS (2012). Asymmetric Dimethylarginine and Reactive Oxygen Species: Unwelcome Twin Visitors to the Cardiovascular and Kidney Disease Tables. Hypertension.

[44] Tripepi G, Raso FM, Sijbrands E, Seck MS, Maas R, Boger R (2011). Inflammation and Asymmetric Dimethylarginine for Predicting Death and Cardiovascular Events in ESRD Patients. Clin J Am Soc Nephrol.

[45] Chan KE, Ikizler TA, Gamboa JL, Yu C, Hakim RM, Brown NJ (2011). Combined angiotensin-converting enzyme inhibition and receptor blockade associate with increased risk of cardiovascular death in hemodialysis patients. Kidney Int.

[46] Grooteman MP, Wauters IM, Teerlink T, Twisk JW, Nube MJ (2007). Plasma dimethylarginine levels in chronic hemodialysis patients are independent of the type of dialyzer applied. Blood Purif.

[47] Busch M, Fleck C, Wolf G, Stein G (2006). Asymmetrical (ADMA) and symmetrical dimethylarginine (SDMA) as potential risk factors for cardiovascular and renal outcome in chronic kidney disease - possible candidates for paradoxical epidemiology?. Amino Acids.

