# Supplementary material for: Angiotensin converting enzyme inhibition increases ADMA concentration in patients on maintenance hemodialysis – a randomized cross-over study
Source: BMC Nephrol. 2015 Oct 22;16:167. doi: 10.1186/s12882-015-0162-x (PMC4618919; doi:10.1186/s12882-015-0162-x)
Supplement: Additional file 1: Figure S1. — CONSORT Flow Diagram. (DOCX 43 kb) [file 12882_2015_162_MOESM1_ESM.docx]

**Supplemental Figure 1: CONSORT Flow Diagram**

## Crossover

## Analysis

## Enrollment

## Allocation

Analysed (n= 15)
♦ Excluded from analysis (n=0)

Lost to follow-up (n=0

Discontinued intervention (n=0)

Crossed-over to receive other study treatment (placebo, ramipril or valsartan, n= 15)

♦ Received allocated intervention (n= 15)

♦ Did not receive allocated intervention (n= 0)

Randomized (n= 15)

Assessed for eligibility (n= 20)

Crossed-over to receive other study treatment (placebo, ramipril or valsartan, n= 15)

♦ Received allocated intervention (n= 15)

♦ Did not receive allocated intervention (n= 0)

Randomly allocated to receive placebo, ramipril, or valsartan (n= 15)

♦ Received allocated intervention (n= 15)

♦ Did not receive allocated intervention (n= 0)

## Follow-Up

Excluded (n= 5)

♦  Not meeting inclusion criteria (n= 3)

♦  Declined to participate (n= 1)

♦  Other reasons (n= 1)
