# Supplementary material for: Angiotensin converting enzyme inhibition increases ADMA concentration in patients on maintenance hemodialysis – a randomized cross-over study
Source: BMC Nephrol. 2015 Oct 22;16:167. doi: 10.1186/s12882-015-0162-x (PMC4618919; doi:10.1186/s12882-015-0162-x)
Supplement: Additional file 2: Table S1. — Baseline characteristics of patients with no history of CKD. (DOCX 14 kb) [file 12882_2015_162_MOESM2_ESM.docx]

| Supplementary Table 1: Baseline characteristics of patients with no history of CKD | | |  |  |  |
| --- | --- | --- | --- | --- | --- |
| Parameter | Placebo  (n=28) | Ramipril  (n=24)l | | | Candesartan  (n=22) |
| Age (yr) | 66.1 ± 2.1 | 64.4 ± 2.1 | | | 67.0 ± 1.7 |
| Gender (male), n (%) | 9 (32.1) | 12 (50) | | | 10 (45.5) |
| Race (caucasian), n (%) | 26 (96.3%) | 24 (100) | | | 22 (100) |
| Past smoking, n (%) | 11 (39.3) | 10 (41.7) | | | 9 (40.9) |
| Hypertension, n (%) | 21 (75) | 17 (70.8) | | | 18 (81.8) |
| Previous ACE inhibitor use, n (%) | 8 (28.6) | 11 (45.8) | | | 8 (36.4) |
| Previous ARB use, n (%) | 6 (21.4) | 2 (8.3) | | | 3 (13.6) |
| BMI (kg/m^2^) | 29.6 ± 1.6 | 29.1 ± 1.2 | | | 30.7± 1.7 |
| Hematocrit (%) | 40.1 ± 0.8 | 41.7 ± 0.8 | | | 40.7 ± 1.0 |
| Data are presented as mean ± SEM |  |  | | |  |
